# Membrane Technology for Valorization of Mango Peel Extracts

**DOI:** 10.3390/foods11172581

**Published:** 2022-08-25

**Authors:** Antónia Macedo, Tânia Gomes, Carlos Ribeiro, Margarida Moldão-Martins, Elizabeth Duarte, Vítor D. Alves

**Affiliations:** 1 Escola Superior Agrária, Polytechnic Institute of Beja, Rua Pedro Soares, Ap. 6158, 7801-908 Beja, Portugal; 2 LEAF—Linking Landscape, Environment, Agriculture and Food, Associated Laboratory TERRA, Instituto Superior de Agronomia, Universidade de Lisboa, 1349-017 Lisboa, Portugal; 3 Instituto Superior de Agronomia, Universidade de Lisboa, Tapada da Ajuda, 1349-017 Lisboa, Portugal

**Keywords:** mango peel, solid-liquid extraction, bioactive components, membrane fractionation, ultrafiltration, nanofiltration

## Abstract

Mango peel is rich in nutritional and functional compounds, such as carbohydrates, dietary fibers, proteins, and phenolic compounds, with high potential to be applied in the food industry. Most of the investigation about recovery of bioactive compounds from fruit bioproducts involves extraction techniques and further separation of target compounds. There is still a lack of information about the potential of membrane processes to recover the nutritive/functional compounds present in aqueous extracts of those bioproducts. This research is addressed to study the performance of ultrafiltration (UF), followed by nanofiltration (NF) of UF permeates, to fractionate the compounds present in aqueous extracts of mango peel. Both UF and NF concentration processes were carried up to a volume concentration factor of 2.0. Membranes with molecular weight cut-offs of 25 kDa and 130 Da were used in the UF and NF steps, respectively. UF and NF concentrates showed antioxidant activity, attributed to the presence of phenolic compounds, with rejections of about 75% and 98.8%, respectively. UF membranes totally rejected the higher molecular weight compounds, and NF membranes almost totally concentrated the fermentable monosaccharides and disaccharides. Therefore, it is envisaged that NF concentrates can be utilized by the food industry or for bioenergy production.

## 1. Introduction

Mango (*Mangifera indica* L.) is a much-appreciated fruit, with a good market acceptance due to its pleasant flavor and texture, being used in a wide variety of foods and products [1]. Mango is the main tropical fruit with the highest volume of production [2], also being one of the five most demanded fruits worldwide [3]. During the industrial processing of mango, peel and stones, the main wastes generated, represent about 35–50% of the fresh fruit [4]. This solid waste is becoming a source of pollution, because of the high volumes produced, and it is a rising concern when landfilled due to its high biodegradability, organic content, and possible environmental impacts [5,6]. Mango peel constitutes around 15–20% of the total fruit weight [4] and its composition can vary with the cultivar, stage of ripening, soil composition, irrigation systems, and climate conditions. However, mango peel has been found to be a good source of important nutrients and functional compounds, including dietary fiber, protein, carbohydrates, phenolic compounds, pectin, carotenoids (mainly trans-β-carotene), tocopherols, ascorbic acid, various vitamins, and minerals [7,8,9,10,11,12]. The antioxidant properties of mango peel, mainly attributed to its content of phenolic compounds bounded to dietary fiber [13,14], makes it available as a supplement and source of fiber in several food formulations, such as bakery products; ice cream; breakfast cereals; beverages; meat products; and as a replacer in cream, cheese, and yogurts [4]. Phenolic compounds, carotenoids, tocopherols, and ascorbic acid from fruit bio-residues can be used as preservatives in natural foods and beverages, because they increase the shelf life of the product, by delaying the formation of off-flavors and rancidity [15,16]. Being an important source of odor-active compounds, mango peel can be used as a flavoring ingredient with applications both in food and cosmetic industries to enhance the mango aroma of the product [17,18,19].

The recovery of high-value components from food biowaste includes several stages, according to the degree of purity to be achieved. Some researchers [20] proposed a “5-Stages Universal Recovery Processing” that generally includes: (i) macroscopic pre-treatment, such as centrifugation, pressing, and size reduction; microfiltration; (ii) separation of macro- and macromolecules, by ultrafiltration and alcohol precipitation; (iii) extraction, using solvent extraction and membrane contactors; isolation-purification, through diafiltration in ultrafiltration mode, nanofiltration, and chromatography; and (iv) product formation or encapsulation. However, depending on the intended purposes, some of these stages can be overcome to decrease complexity and cost. In industry, solid-liquid extraction is mainly used for the recovery of anthocyanins by using hydroalcoholic mixtures, where ethanol is the alcohol [20,21]. High concentrations of bioactive compounds in fruit and vegetable extracts were obtained after extraction with hot water or a hydroalcoholic mixture [22].

Membrane separation processes have been applied in the recovery of fruit biowaste components, as is the case of ultrafiltration and nanofiltration, for clarification, separation/concentration, or purification purposes. To clarify kiwi juice, ultrafiltration (UF) was investigated using tubular membrane modules [23,24]. The resulting permeate was subjected to a concentration step by osmotic distillation until obtaining a final concentrate with a total soluble solid of 62–65 °Brix, which could be used for the preparation of fiber-enriched beverages (nectars). Nanofiltration (NF) was used to reduce the sugar content of grape must in the production of alcoholic wines [25]. The performance of two nanofiltration membranes, DK and DL commercialized by Osmonics, to concentrate grape must for increasing the sugar concentration for wine production was investigated by some authors [26]. They concluded that both membranes showed a high rejection of sugars (77–97%) and polyphenols (70–94%), proving suitable for that purpose. Nanofiltration membranes with molecular weight cut-offs (MWCO) in the range 150–350 Da, were used in the treatment of aqueous extracts of grape pomace, obtained by pressing and distillation [27]. The authors observed high rejections of phenolic compounds and sugars, with the content of phenolic compounds in the retentates having increased by a factor of three to six, in relation to the feed.

The performance of nanofiltration for the separation and concentration of bioactive compounds present in elderberry (*Sambucus nigra* L.) juice, to obtain fractions to be used in the formulation of functional foods, was also evaluated [28]. The retentates obtained with NP030 membranes, commercialized by Microdyn-Nadir, showed the highest antioxidant activity, so the investigators considered that those fractions were very interesting for the intended purpose.

Given the effectiveness of membrane processes in separating compounds from fruit aqueous extracts and juices, the main goal of the present work is to valorize mango peel, with the application of UF and NF processes to fractionate and concentrate mango peel aqueous extracts. The overall approach includes the pretreatment of mango peel through size reduction, followed by hydrothermal aqueous extraction, ultrafiltration of aqueous extracts, and nanofiltration of the corresponding permeates. The fractions obtained were characterized in terms of chemical composition and antioxidant activity, as well as molecular size distribution of the carbohydrates, in order to assess their potential industrial applications (e.g., food products or bioenergy production).

## 2. Materials and Methods

### 2.1. Pretreatment of Mango Peels and Production of Aqueous Extracts

Mango peels of different varieties were supplied by local market at Beja, in Portugal. Immediately after reception, mango peels were washed with cold water, the superficial water was removed with a cotton cloth, stored in plastic bags, each with 500 g and frozen at a temperature of −18 °C, until processing. To prepare the aqueous extracts, mango peels were thawed in a refrigerator at 4 °C, weighed, and subjected to size reduction by milling, to homogenize and allow a better recovery during the extraction process as indicated by several authors [20,21]. Hot water was added to this preparation, at a temperature of 70 °C [29,30], keeping a solid-to-liquid ratio 1:10 (1 kg of fresh mango peel/10 L of water), because this proportion was considered the most suitable ratio to achieve a high extract yield and a reduced operating cost for these kind of samples [31]. The heating of aqueous extracts was carried out in a water bath with stirring, at an orbital speed of 1000 min^−1^, for 75 min, a time considered optimum in the preparation of aqueous extracts from fruit bioresidues [29]. The extracts obtained were then filtered through cotton cloths to separate the liquid and solid fractions. The filtrates were used as ultrafiltration feeds.

### 2.2. Characterization of Mango Peels

Mango peels were analyzed for the following parameters: moisture of the samples was determined gravimetrically, according to the official method AOAC 920.51 for fruit and fruit products [32], in three replicates; pH, by the potentiometric method using the potentiometer Methrom 744 pH Meter; titratable acidity, according to the AOAC 943.03 official method for fruits and fruit products [33]; water activity was measured directly using the hygrometer HP23-AW-A; the content of soluble solids, expressed in degree brix (°Brix) using the refractometer, Bellingham & Stanley Ltd. RFM 330; crude protein was determined by the Kjeldahl method according to the AOAC 920.152 official method for fruit products [34]; crude fat was extracted and then quantified by using the official method (AOAC, 1984) [35], with prior hydrolysis of the sample and using a Tecator equipment, consisting of the Soxtec System HT extraction unit and the heating unit 1043; ash was analyzed based on the AOAC(1990) method [36]; the determination of the total, soluble, and insoluble dietary fiber contents were carried out according to the AOAC 991.43 method [37]; the carbohydrate content, HC, was determined by calculation according to the equation: HC = 100 − (fat + crude protein + fiber + ash) [1].

### 2.3. Characterization of Aqueous Extracts

Aqueous extracts from mango peels, concentrates, and permeates of ultrafiltration and nanofiltration processes, were analyzed in terms of the following parameters: pH; total solids; °Brix; ash; total protein; fat; total carbohydrates; monosaccharides and disaccharides; total soluble phenols; and antioxidant activity. The determination of total carbohydrates was performed by the spectrophotometric method of Dubois, with some modifications [38]. A volume of 1 mL of diluted sample was introduced into a test tube to which was added 1 mL of 5% aqueous phenol solution. This mixture was mixed vigorously by vortexing. Then, 5 mL of concentrated sulfuric acid (d = 1.84) was added and mixed. This mixture was allowed to stand for 10 min, then vortexed again and cooled in a water bath at a temperature between 20–25 °C. After waiting 5 min for color development, absorbance was measured with a spectrophotometer at λ = 490 nm. The calibration curve was previously prepared in the same way using a set of standard glucose solutions, with concentrations between 5 and 60 mg/L. The monosaccharides glucose, galactose, and fructose, and the disaccharides, sucrose and maltose, were analyzed by High Performance Liquid Chromatography/Ion Chromatography (HPLC/IC) [39]. CarboPac PA10 column (Dionex) equipped with an amperometric detector was used. The analysis was performed at 30 °C, with sodium hydroxide (NaOH 4 mmol/L) as eluent, at a flow rate of 0.9 mL/min. Glucose, galactose, fructose, sucrose, and maltose (Panreac Quimica SAU, Barcelona, Spain) were used as standards (0.006–0.2 g/L). Total phenolic content was evaluated using the Folin-Ciocalteau method, according to the following procedure: 20 μL of diluted aqueous extract was mixed with 100 μL of Folin-Ciocalteau reagent diluted 1:10 and 75 μL of sodium carbonate (75 g/L) in a well of the microplate. After 2 h in the dark at room temperature, absorbance was measured at 740 nm on a Fluostar Optima microplate reader, BMG Labtch, in a 96-well clear flat-bottomed microplate [40]. Gallic acid monohydrate was used as a standard in the range 2–10 mg EAG/100 mL to obtain a calibration curve. The antioxidant activity was evaluated using the Ferric Reducing Antioxidant Power (FRAP) method. In this case, an aliquot of 20 μL of aqueous extract was mixed with 30 μL of water in a microplate well. A volume of 200 μL of the FRAP reagent prepared daily was added, consisting of 10 volumes of 300 mmol/L of acetate buffer (pH 3.6), 10 volumes of FeCl_3_ at 20 mmol/L, plus 1 volume of 2,4,6-tripyridyl-s-triazine (TPTZ) diluted in 40 mmol/L hydrochloric acid, with the ferric TPTZ complex being reduced to its iron (II) form by the antioxidants. Absorbances were measured on a Fluostar Optima microplate reader, BMG Labtch, in a 96-well clear flat-bottomed microplate, using a calibration curve with standard solutions of Trolox (10–150 μmol/mL) and water as a blank. The antioxidant capacity of the samples was expressed as Trolox equivalents (TE) in μmoL/100 mL [40].

### 2.4. Molecular Weight of Polysaccharides in UF/NF Fractions

The application of Gel Permeation Chromatography/Size Exclusion Chromatography (GPC/SEC) was carried out using a Phenomenex Polysep P500+P3000 GFC column of 300 × 7.8 mm, 250 Da–2 MDa, with a liquid flow rate of 0.6 mL/min (DMAC/LiCl 0.5%, *w/v* solution), at 25 °C, using an injection volume of 50 μL and a RID detector (HP 1047A). The samples were analyzed after their filtration through a NY filter, with pore size of 0.22 μm. The calibration curve was built with eight Pullulan standards, in the range 642 kDa–6.3 kDa (SHODEX). The number average molecular weight, M_n_, of the samples was determined based on Equation (1):

(1) $$M_{n}=\frac{\sum N_{i}M_{i}}{\sum N_{i}}$$

where M_i_ is the molecular weight of a polymer chain; N_i_, the number of chains with that molecular weight.

Equation (2) was used to determine the weight average molecular weight, M_w_:

(2) $$M_{w}=\frac{\sum N_{i}M_{i}^{2}}{\sum N_{i}M_{i}}$$

The polydispersity index, PI, was calculated based on Equation (3):

(3) $$\mathrm{PI}\;=\frac{M_{w}}{M_{n}}$$

### 2.5. Membranes and Experimental Set-Up

The ultrafiltration (GR60PP) and nanofiltration (NF) membranes used are commercialized by the company Alfa-Laval, Portugal. These membranes are asymmetric, with a surface membrane area of 0.018 m^2^. Their main characteristics are presented in Table 1.

**Figure 1:**
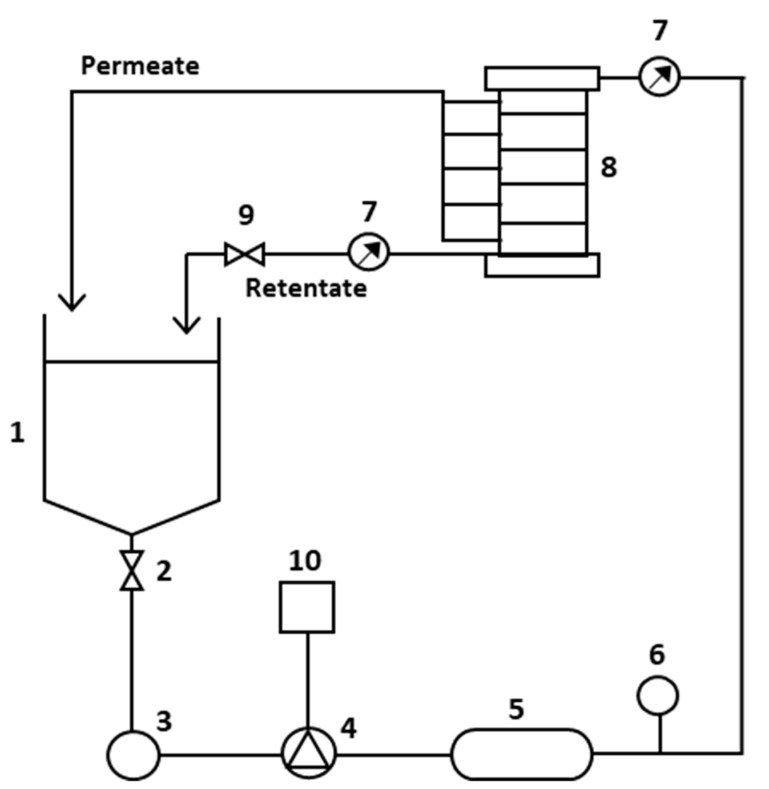
Schematic illustration of Lab-unit M20 (adapted from the operating manual [42]). (1) Feed tank, (2) valve, (3) filter, (4) cross-flow pump, (5) heat exchanger, (6) dampener, (7) pressure gauge, (8) membrane module, (9) pressure control valve, (10) cross-flow pump control.

The filtration experiments were performed in a plate and frame module (LabUnit M20, DSS Alfa Laval, Nakskov, Denmark), with the experimental set-up shown in Figure 1.

It is a versatile installation that allows operating with microfiltration, ultrafiltration, nanofiltration, and reverse-osmosis membranes in batch mode. It is equipped with two sets of pressure gauges, one of them to work at low pressures, until 10 bar, as usual in microfiltration and ultrafiltration processes, and another for high pressures, until 100 bar, for using in nanofiltration and reverse osmosis processes. In all the experiments carried out, both in ultrafiltration and nanofiltration modes, the total surface membrane was 0.072 m^2^, corresponding to four membranes.

### 2.6. Experimental Design

#### 2.6.1. Membrane Cleaning

A cleaning-in-place cycle was performed after each filtration experiment to remove fouling and recover as much as possible the membranes hydraulic permeability, and to prevent microbiological contamination. The cycle involves several steps, as presented in Table 2.

This cycle was carried out respecting the limits of pressure, temperature, pH, and concentrations of cleaning and disinfection agents, according to the type of membrane. All these cleaning steps were carried out with the membrane installation operating in total recirculation mode, i.e., the retentate is fully recirculated to the feed tank. The transmembrane pressure used was 1 bar for ultrafiltration membranes and 8 bar for nanofiltration membranes, keeping a feed circulation flow rate of 10.0 L/min, at room temperature. After the permeation of each solution, membranes were rinsed twice with deionized water to remove any residues that may have remained on the membranes and in the installation’s pipes.

#### 2.6.2. Determination of Hydraulic Permeability of Membranes to Deionized Water

The solvent hydraulic permeability of a given membrane is characteristic of it and its value is used as a reference to evaluate the cleaning process efficiency and possible fouling after the essays with real solutions. The experimental determination of the hydraulic permeability of ultrafiltration membranes was carried out by measuring the permeate fluxes of deionized water at 25 °C and different transmembrane pressures in the range 0.5–4 bar, with a feed circulation flow rate of 10.0 L min^−1^ that corresponds to a feed tangential velocity of 0.92 ms^−1^. Between every two pressures, permeate fluxes were allowed to stabilize for 30 min. Experimental determination of the hydraulic permeability of nanofiltration membranes was carried out in a similar way but measuring the permeate fluxes to deionized water in the range of transmembrane pressures of 8–20 bar. The experimental permeate fluxes, J_w_, were calculated based on Equation (4):

(4) $$J_{w}=\frac{V_{p}}{A\;\times \;t}$$

where J_w_ is the volumetric permeate flux (L h^−1^ m^−2^), V_p_ is permeate volume (L), t the time (h) needed to collect the permeate volume V_p_, and A is the membrane area (m^2^).

For the permeation of a pure solvent across a membrane, permeate fluxes are proportional to the applied transmembrane pressure, in accordance with Equation (5) [43]:

(5) $$J_{w}=\frac{L_{p}}{\mu }\times \Delta P$$

where L_p_ is the membrane intrinsic permeability that depends only on its morphological characteristics; μ is the viscosity of the permeate; ΔP is the transmembrane pressure applied (bar); and L_p_/μ, is the membranes hydraulic permeability (L h^−1^ m^−2^ bar^−1^).

#### 2.6.3. Ultrafiltration Experiments of Aqueous Extracts

Ultrafiltration experiments were first carried out in total recirculation mode, where the retentate and permeate were recirculated to the feed tank, so the concentration of the components in the feed tank remained constant. These tests were performed with the aqueous extracts of mango peels using membranes GR60PP, with a membrane area of 0.072 m^2^, at transmembrane pressure in the range 1–4 bar, at a feed circulation flow rate of 10.0 L min^−1^, and a temperature of 25 °C. The permeate fluxes were measured sequentially at different transmembrane pressures after stabilization at each pressure for 30 min and calculated according to Equation (4).

Based on the results obtained, the most suitable transmembrane pressure was selected to carry out the ultrafiltration of aqueous extracts in concentration mode (ΔP = 2 bar). In this case, the permeate was collected continuously and only the retentate was totally recycled to the feed tank. These experiments were performed with a feed circulation flow rate of 10 L min^−1^ (feed velocity of 0.94 ms^−1^), and temperature of 25 °C, until a volumetric concentration factor (VCF) of 2.0 was achieved. The volumetric concentration factor (VCF) was calculated with Equation (6):

(6) $$\mathrm{VCF}=\frac{V_{\mathrm{feed}}}{V_{\mathrm{conc}}}=\frac{V_{\mathrm{feed}}}{V_{\mathrm{feed}}-V_{p}}$$

where V_feed_ is the initial volume of feed; V_conc_ is the volume of the concentrate; and V_p_ is the volume of permeate.

Samples of the final concentrates and of the corresponding permeates were collected for determining the apparent rejection coefficients, R_i_, calculated with Equation (7) [43]

(7) $$R_{i}=\frac{C_{i,\mathrm{conc}}-C_{i,p}}{C_{i,\mathrm{conc}}}\times 100$$

where C_i,conc_ is the bulk concentration of component i in the concentrate, and C_i,p_ is the bulk concentration of the component i in the corresponding permeate. After each experiment, membranes were subjected to the cleaning and disinfection procedure described in Table 2 and its hydraulic permeability to pure water was then verified as described in Section 2.6.2.

#### 2.6.4. Nanofiltration Experiments of Ultrafiltration Permeates

The ultrafiltration permeates were processed by nanofiltration in total recirculation mode, with the NF membrane, at transmembrane pressures between 8–20 bar, maintaining a feed circulation flow rate of 10.0 L min^−1^ and a temperature of 25 °C. The stabilization time at each pressure was 30 min and permeate fluxes were experimentally determined based on Equation (4). Afterwards, the permeates of ultrafiltration were concentrated (up to VCF = 2.0) by nanofiltration operating in concentration mode, with ΔP = 20 bar, the maximum transmembrane pressure used in total recirculation experiments and for which the highest permeate flux was reached. The feed circulation velocity was kept at 0.94 ms^−1^ and temperature at 25 °C. Samples of concentrate and corresponding permeate were taken for analysis at VCF = 2.0. Again, after each experiment, membranes were subjected to the cleaning and disinfection procedure described in Table 2, and their hydraulic permeability was checked as described in Section 2.6.2.

#### 2.6.5. Statistical Analysis

Statistical analysis was performed using the R program, version 4.1.3. It included the determination of descriptive statistic parameters, such as average and standard deviation values, as well as to estimate the parameters of linear regressions for confidence intervals of *p* < 0.05.

## 3. Results and Discussion

### 3.1. Physicochemical Characterization of Mango Peels

The physicochemical characterization of mango peels and of the corresponding aqueous extracts obtained for the solid/liquid ratio of 1:10 is shown in Table 3, where the mean values ± standard deviation for three replicates of each parameter/sample are presented.

Mango peel has an accentuated acid behavior, with a pH = 4.87 ± 0.03, which can help to prevent the proliferation of molds and yeasts, thus facilitating their conservation and storage. However, an a_w_ of 0.92, higher than the limit of 0.90, should be of concern to avoid any contamination during their manipulation [44]. The predominant components in mango peel are carbohydrates, fiber, protein, ash, and fat, which agrees with that described in literature [12]. It was observed that the chemical composition of mango peel varied depending on the cultivar, both fresh and ripe, as follows: moisture, between 62% and 83%, with dry matter consisting of carbohydrates (>70%), total dietary fiber (35.5–78.3%), protein (1.5–6.6%), ash (1.2–4.2%), fat (1.6–3.7%), in addition to vitamins, phenolic compounds, carotenoids, and volatile compounds. The results obtained in this study can, in general, be included in the same range of all the parameters, except for the fat content whose value was below the lower limit for this parameter. However, other authors obtained closer values for fat in mango peel, 0.84% for (*Mangífera indica* L. cv. Tommy Atkins) [45]. The raw fiber contents are lower than the minimum limit indicated for the total dietary fiber in that study [12], possibly because in the analysis of dietary fiber, other compounds are accounted for, such as resistant starch and pectins [46].

### 3.2. Physicochemical Characterization of Aqueous Extracts

The results of the aqueous extracts obtained by the authors highlight that the dry matter is mainly composed of carbohydrates, followed by minerals (Table 3). The content of total soluble phenols obtained, around 62.5 mg EAG/g of dried peel, is like that observed by other authors [1], which was 64.8 mg EAG/g of dried peel. This small difference can be due not only to the cultivar used in their work (*Mangifera indica* L. var *Sugar*), but also to the different extraction process that was carried out, namely a sequential extraction with an acidified methanol-water solution, followed by an acetone-water solution and centrifugation for the separation of the supernatant extracts. Then, the supernatants obtained were mixed and submitted to the Folin-Ciocalteau method. Comparing the total soluble phenols of mango peels with other fruit biowaste, such as papaya (0.6 mg EAG/g of peel), orange (2.2 mg GAE/g of peel), and passion fruit (0.7 mg GAE/g of peel), it can be concluded that mango peel is a promising source of phenolic compounds [1,12].

The antioxidant capacity of the aqueous extract of 81.6 μmol TE/100 mL is within the average values determined by some authors [46] and, as expressed in a dry basis, 46.1 μmol TE/g of mango peel is close to the range of values determined by other authors for extraction with water [47]. However, the antioxidant activity of the present extract is roughly half of that referred in another study, where the extraction was done with 80% methanol [48,49]. This fact may be attributed to the use of organic solvents with greater affinity for the solutes to be separated, which may increase the extraction yield. This procedure is not possible when extracts are intended to be used in the food industry. One possible way to improve extraction yield could be to use an ethanol/water mixture as solvent [21].

### 3.3. Ultrafiltration of Aqueous Extracts

#### 3.3.1. Ultrafiltration with Total Recirculation

**Figure 2:**
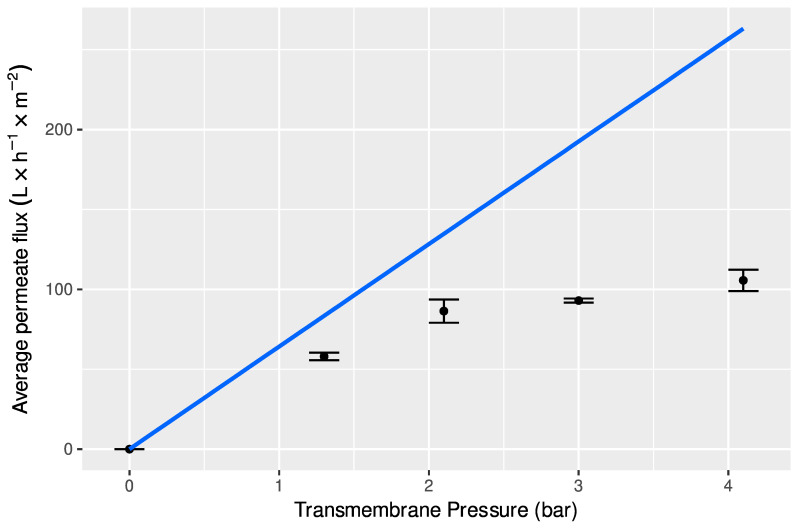
Variation of water fluxes (line) and permeate fluxes of aqueous extracts from mango peels (symbols), with transmembrane pressure, obtained with membranes GR60PP at v = 0.91 ms^−1^, T = 25 °C, and membrane area = 0.072 m^2^.

The comparison between average water permeate fluxes, J_w_ (line) and average (± standard deviation) extract permeates fluxes, J_p_, in the same range of transmembrane pressure and similar experimental conditions, such as feed circulation velocity of 0.94 ms^−1^ and temperature of 25 °C, is shown in Figure 2.

It can be observed that the permeate fluxes obtained with the aqueous extracts of mango peel increased linearly with transmembrane pressure in the region of low pressures, up to approximately 2 bar, and then there was an approach to a plateau for pressures higher than 2.0 bar. This flux pattern indicates that permeate fluxes are controlled by the pressure in the low-pressure region, while in the high-pressure zone, the main mechanism that controls the UF process is mass transfer [50]. Comparing aqueous extract permeate flux with water flux for lower pressures, it can be seen that permeate flux is closer to water flux, reaching about 70% of the later, for a pressure of 2.0 bar. However, permeate flux sharply moves away from water flux for higher pressures, being around 50% of water fluxes for a transmembrane pressure of 3.0 bar, and about 30% for a pressure of 4.2 bar. This behavior is attributed to the effect of polarization concentration phenomena, which is usually negligible for lower pressures (lower fluxes), and becomes increasingly important when pressure increases [51,52,53]. The increase of transmembrane pressure, and therefore of the permeate flux, leads to the growth of the thickness of the polarization layer next to the membrane surface, which offers an increasing resistance to the permeate transfer [43]. Therefore, to carry out the concentration process of the aqueous extracts of mango peels by ultrafiltration, the selected transmembrane pressure was 2.0 bar, because it was the highest pressure in the linear region that led to higher permeate fluxes.

#### 3.3.2. Ultrafiltration in Concentration Mode

Concentration experiments were carried out with GR60PP membranes, at the selected transmembrane pressure of 2.0 bar, at a feed circulation velocity of 0.94 ms^−1^, a temperature of 25 °C, and with a membrane area of 0.072 m^2^. Various consecutive UF concentration essays of aqueous extracts were performed in those same conditions. During the first ultrafiltration essay with mango peel aqueous extracts until a VCF = 2.0, permeate fluxes varied from 80 to 60 L h^−1^ m^−2^ and the same variation was observed during the second UF experiment. The decrease of permeate fluxes during the runs can be attributed to membrane fouling, likely caused by adsorption of phenolic compounds and proteins on membranes surface [43,51]. This fact was confirmed by determining the hydraulic permeability of the membranes after carrying out the cleaning and disinfection cycle shown in Table 2. After the first ultrafiltration essay and having undergone a complete cleaning and disinfection cycle, the hydraulic permeability of the membranes was 92% of its initial value. After the second ultrafiltration essay with the same membranes, the decrease of hydraulic permeability of membranes was higher, being about 68% of its initial value, though remaining stable in the following ultrafiltration tests. These results allow to conclude that irreversible membrane fouling was increasing along the several experiments [43,51]. A possible way to overcome this aspect could be to use a membrane of a similar cut-off, but made of a more hydrophilic material, which is generally less prone to adsorption of organic materials, especially molecules such as proteins and phenolic compounds [52,54].

#### 3.3.3. Fractionation of Extracts by UF

To study the extracts fractionation by UF membranes, apparent rejection coefficients were determined based on Equation (7), for the VCF = 2. The obtained results are shown in Table 4.

The rejection coefficient of total carbohydrates was 22%, which means that most of these compounds were recovered in ultrafiltration permeates. Since UF membranes have a MWCO of 25 kDa, most of the retained carbohydrates should have molecular weights higher than 25 kDa and are probably polysaccharides. This was confirmed by the distribution of molecular weights from GPC/SEC analysis, presented in Section 3.5. The rejection of total soluble phenolic compounds was about 35%, which was higher than expected, as the identified phenolic compounds in mango peels are xanthones, benzophenones, gallic acid, gallates, gallotannins, flavonoids, cinnamic acids, and derivatives [12], which have lower molar masses than the molecular weight cut-off of the membranes. The greater retention of these phenolic compounds may be due to their interactions with the polysaccharides present. The high rejection of the antioxidant capacity, about 75%, is in line with the rejection of phenolic compounds. Some researchers found that the antioxidant activity of mango peel was correlated with the presence of bioactive compounds, mainly phenolic compounds [55], generally bound in large amounts to dietary fiber [14,56]. Regarding sugars with lower molecular weights, such as the monosaccharides and disaccharides, it can be observed that their rejection varied in the range 1.0–22%. These values are higher than expected, considering the MWCO of membranes and that the main separation mechanism in ultrafiltration is usually molecular exclusion. These results can be attributed to the membrane fouling, which may have been caused by the formation of a second dynamic membrane built with the macromolecules present, which led to an increase in the rejections of the compounds of lower molecular mass [57]. The rejection coefficients of total protein and fat are not shown in Table 4, because their concentrations in permeates were under the detection limits of the analytical methods used.

### 3.4. Nanofiltration of Ultrafiltration Permeates

#### 3.4.1. Nanofiltration with Total Recirculation and Concentration Modes

**Figure 3:**
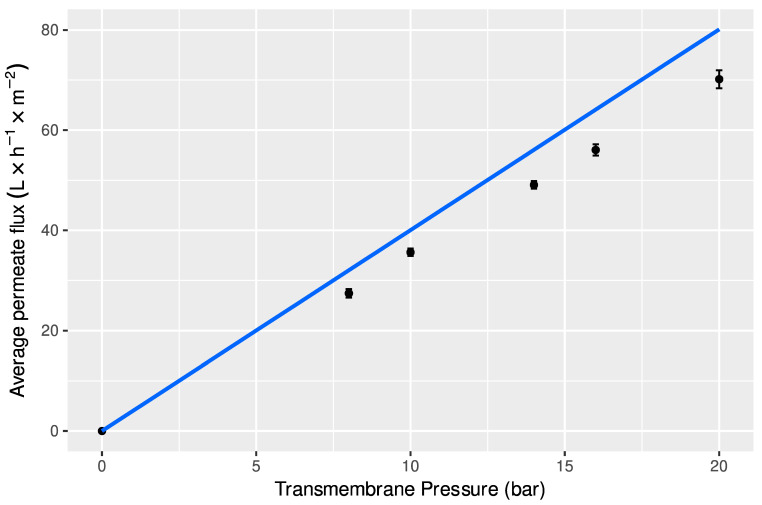
Variation of water fluxes (line) and permeate fluxes of ultrafiltration concentrates (symbols) with transmembrane pressure, during nanofiltration of ultrafiltration concentrates with membranes NF, at v = 0.91 ms^−1^, T = 25 °C, and membrane area = 0.072 m^2^.

Figure 3 shows the variation of average water fluxes, represented by the line, and average permeate fluxes (±standard deviation) obtained during the nanofiltration process of ultrafiltration permeates, with transmembrane pressure.

It can be observed that permeate fluxes increased linearly with the applied transmembrane pressure, up to a pressure of 20.0 bar. This behavior is usual in nanofiltration, because in this process permeate fluxes are lower than those observed in ultrafiltration, and then, the rapid accumulation of retained solutes near the membrane surface responsible for the polarization concentration phenomena is less important. However, permeate fluxes are lower than water fluxes in all the range of pressures studied, which can be attributed mainly to the osmotic pressure of the samples, due to the retention of lower molar mass solutes, such as monosaccharides and disaccharides [43]. The high rejection coefficients of these simple sugars, as displayed in Table 5, confirms the influence of osmotic pressure difference between feed and permeate. This phenomenon leads to the lowering of the effective transmembrane pressure, causing the decline of permeate fluxes [52,58]. Since the highest permeate fluxes were obtained at the maximum pressure studied, the nanofiltration tests in concentration mode were carried out at this pressure, keeping the feed circulation velocity at 0.92 ms^−1^ and temperature at 25 °C.

During the concentration process until a VCF = 2.0, the average permeate fluxes were constant with a value around 58 ± 5 L h^−1^ m^−2^, which indicates that during these essays the fouling phenomena was not relevant, which means that these membranes can be used to concentrate these samples until higher VCF values, [59,60]. Besides, after NF experiments with the samples, the hydraulic permeability of membranes was recovered after cleaning, being about 97% of the initial hydraulic permeability of the new membranes.

#### 3.4.2. Rejection Coefficients of Compounds Fractionated by NF Membranes

The rejection coefficients of total carbohydrates, monosaccharides, and disaccharides analyzed, as well as of total soluble phenols and antioxidant capacity, are shown in Table 5.

It can be observed that membranes NF were suitable for the recovery and concentration of all the monosaccharides and disaccharides present, with quantitative rejections between 98 and 100%, except for glucose, in which rejection was a little lower, around 82%. The rejection coefficients of both monosaccharides and disaccharides are very similar. These results are in agreement with those obtained in other works, where several NF membranes made with different materials and MWCO values were used to separate saccharose from reducing sugars (glucose/fructose) [61,62]. At room temperature, with membranes made of the same material and MWCO of those of the present work, those authors presented rejection coefficients for saccharose and glucose/fructose, higher than 90% and 80%, respectively. The authors attributed these results to the fact that in NF processes the separation of neutral organic compounds is mainly governed by the molecular exclusion mechanism [62]. Since the MWCO of NF membranes (about 160 Da) and the molecular weight differences between saccharose and glucose/fructose/galactose is only 162 Da, and their stokes radius differ around 0.1 nm [63], it is difficult to separate those sugars only based on size exclusion, under the operating conditions used.

Due to the high rejection of phenolic compounds, about 92%, and the related antioxidant activity, the nanofiltration concentrates seem to be very interesting for future uses in functional foods. Similar rejection of phenolic compounds (95.7%) and antioxidant activity (90%) were found by other researchers during the recovery of polyphenols and organic acids from red wine lees, using NF membranes with MWCO of around 200 Da [64]. For further separation between phenolic compounds and monosaccharides/disaccharides, other membranes or operating conditions should be tested to find barriers with the appropriate selectivity.

### 3.5. Molecular Weight Distribution of Compounds Separated by UF/NF

The molecular weight distribution of polysaccharides, monosaccharides, and disaccharides separated by UF/NF and analyzed by GPC/SEC, is shown in Table 6. The parameters evaluated were Mn, Mw, and PI.

It can be observed that, both in the UF feeds (aqueous extracts) and in the respective concentrates, two distinct ranges of average molecular weight values were detected. One of them corresponds to lower molecular mass compounds, between 371 and 511 Da, and the other to much higher molecular mass molecules, between 33,843 and 89,281 Da. The first can be attributed to simple carbohydrates, such as mono and disaccharides, which was confirmed by the respective rejection coefficients obtained from the results of HPLC/IC (Table 4). Some deviations in the Mw’s obtained in relation to the respective molecular weights of the monosaccharides and dissacharides can be attributed to the fact that the calibration curve was obtained with standards between 642 kDa and 6.3 kDa, a higher molecular weight range than the molar masses of these compounds, which may have led to some inaccuracy in these values.

The second range of average molecular weight values is attributed to polysaccharides that constitute the dietary fiber. These compounds are not present in the UF permeates and, consequently, in the NF fractions. Therefore, the ultrafiltration membranes used (GR60PP) were able to totally retain the higher molecular weight carbohydrates present in the aqueous extracts of mango peel. In the UF permeates, only one range of average molecular weight is detected, corresponding to lower molecular mass compounds (Mw = 472 Da). In fact, the UF process has been successfully used to separate polysaccharides in different fractions using membranes with different MWCO values. As examples, Chen et al., 2021 [65] isolated pectic polysaccharides from red pitaya peel extracts into three fractions (<50 kDa, 50–100 kDa, and >100 kDa) using membranes with 50 and 100 kDa MWCO. In addition, primary cell wall polysaccharides from aqueous extract of buriti fruit pulp were purified by sequential UF, obtaining two homogeneous fractions (M_w_ of 126 kDa and 20 kDa) [66].

Regarding nanofiltration fractions, both in feed, concentrates, and respective permeates, there is only one family of low molecular weight compounds. It is attributed to the monosaccharides or disaccharides analyzed by HPLC/IC (Table 4), and also to soluble phenolic compounds, with similar molecular weights, taking into account the rejection coefficients presented for UF (Table 4) and NF (Table 5) processes. NF process has been used with success in the recovery of phenolic compounds from various sources, such as pomegranate husk [67], olive mill wastewaters, and artichoke wastewaters [68]. In NF feeds and concentrates, Mw varies between 350 and 436 Da and, in permeates, between 251 and 261 Da. Regarding sugars, those present in NF permeates should be mainly glucose and fructose, which were not totally rejected by the NF membranes, according to the rejection coefficients presented in Table 5. Depending on the NF membrane MWCO, monosaccharides may still be transferred to the permeate [68].

## 4. Conclusions

Mango peel aqueous extracts were processed by ultrafiltration (UF), followed by nanofiltration (NF) of the respective permeates. This methodology allowed a total separation between polysaccharides and mono/disaccharides (glucose, fructose, galactose, and saccharose) present in aqueous extracts. In addition, both UF and NF concentrates presented antioxidant activity, attributed to the simultaneous retention of phenolic compounds (35% and 92% for UF and NF, respectively). As such, these concentrates may find application in the formulation of functional food products. NF concentrates may also be used for bioenergy production, as they contain almost all the fermentable sugars present in the extract, namely glucose, fructose, galactose, and saccharose. However, since they also contain phenolic compounds, it should be assessed if their presence can affect bioenergy production. Regarding NF permeates, as they contain very low concentrations of monosaccharides and soluble phenolic compounds, they may be reused as water for the extraction process of mango peels, minimizing water consumption. To implement a full circular approach for the proposed mango peels valorization process, it is fundamental to find an end use for the solid fraction discharged during the preparation of aqueous extracts. This solid fraction may be directed for ruminant feed, feedstock for biofuels and platform chemicals, or to be used as a source of nitrogen for soils.

## Figures and Tables

**Table 1:** Membrane characteristics.

Membrane	Material ^b^	MWCO ^a^ (Da)	Max. Temperature ^b^ (°C)	pH Range ^b^	Pressure Range ^b^	Hydraulic Permeability ^d^ (Lh^−1^ m^−2^ bar^−1^)
GR60PP (UF)	Polysulfone	25,000 ^b^	75	2–10	1–10	63.68 ± 2.94
NF	Polypiperazine	130 ^c^	50	3–9	15–35	3.54 ± 0.20

^a^ Molecular weight cut off; ^b^ Indicated by the manufacturer; ^c^ Macedo et al., 2018 [41]; the retention of a solution of MgSO_4_ at 2000 mg/L, by NF membrane, is >99%, at 9 bar and 25 °C, as indicated by the manufacturer; ^d^ measured experimentally in the present work as the slope of permeate flux as a function of transmembrane pressure as described in Equation (5).

**Table 2:** Cleaning and disinfection processes used with UF and NF membranes.

Solution Type	Solution	Time (Min)	Objective
Cleaning			
Alkaline conditions	Sodium hydroxide solution, 0.05% (*w*/*v*)	15	Removal of organic compounds (proteins, fat, sugars)
	Na-EDTA ^a^ solution, 0.2% (*w*/*v*)	15	
Acid conditions	Nitric acid solution, 0.25% (*w*/*v*)	15	Removal of minerals and salts
	Monohydrate citric acid solution, 0.5% (*w*/*v*)	15	
Disinfection	Hydrogen peroxide solution, 1000 ppm	30	Elimination of microorganisms

^a^ Na-EDTA—ethylenediaminetetra-acetic acid, sodium salt.

**Table 3:** Physicochemical and functional characterization of mango peels and aqueous extracts (solid/liquid ratio of 1:10).

	Samples	
Parameter	Mango Peels	Aqueous Extracts (1:10)
pH (T = 25 °C)	4.87 ± 0.03	4.12 ± 0.24
Titration acidity (% citric acid)	0.02 ± 0.001	-
Moisture (% *w*/*w*)	82.29 ± 0.12	-
a_w_	0.92 ± 0.01	-
°Bx (total soluble solids)	15 ± 0.58	1.0 ± 0.10
Total protein ^a^ (%*w*/*w*)	3.27 ± 0.43	9.62 ± 0.20
Fat ^a^ (% *w*/*w*)	0.62 ± 0.11	0.02 ± 0.01
Ash ^a^ (% *w*/*w*)	3.69 ± 0.07	12.02 ± 0.83
Raw fiber ^a^ (%*w*/*w*)	11.39 ± 0.23	-
Carbohydrates (% *w*/*w*)	81.03 ^a,b^ ± 0.05	77.48 ± 2.92
Total soluble phenols ^a^ (mg EAG/g of mango peel) ^c^	-	62.5 ± 2.8
Antioxidant capacity ^a^ (μmol TE/g of mango peel)	-	46.1 ± 1.6 (81.6 μmol TE/100 mL)

^a^ Concentration in a dry basis; ^b^ Calculated by the difference to 100 with the other components; ^c^ EAG: equivalents of acid gallic; Total solids content in aqueous extracts is 1.04% *w*/*w*.

**Table 4:** Rejection coefficients of compounds by ultrafiltration membranes (GR60PP), for a VCF = 2.

Rejection Coefficients (%)							
Total Carbohydrates	Glucose	Galactose	Fructose	Saccharose	Ash	Total Soluble Phenols	Antioxidant Capacity
22.4 ± 2	22 ± 3	4 ± 1	14 ± 2	1 ± 0.1	2.1 ± 0.1	35.0 ± 2	75.0 ± 4

**Table 5:** Rejection coefficients for compounds fractionated by NF membranes for a VFC = 2.0.

Rejection Coefficients (%)						
Total Carbohydrates	Glucose	Fructose	Galactose	Saccharose	Total Soluble Phenols	Antioxidant Capacity
99 ± 2	82± 1	98 ± 1	100 ± 0	100 ± 0	92± 3	99 ± 2

**Table 6:** Molecular weight distribution of compounds fractionated by UF/NF.

Sample	^a^ M_n_ (Da)	^b^ M_w_ (Da)	^c^ PI
Feed (UF)	⌈24,051−32,646⌉	⌈33,843−89,281⌉	⌈1.41−2.74⌉
	⌈267−269⌉	⌈371−501⌉	⌈1.38−1.60⌉
Concentrate (UF)	⌈29,444−29,413⌉	⌈66,532−74,568⌉	⌈2.26−2.53⌉
	⌈267−314⌉	⌈391−511⌉	⌈1.47−1.63⌉
Permeate (UF)	⌈263−299⌉	⌈350−472⌉	⌈1.33−1.58⌉
Feed (NF)	⌈263−288⌉	⌈350−436⌉	⌈1.33−1.52⌉
Concentrate (NF)	260	355	1.37
Permeate (NF)	⌈235−244⌉	⌈251−261⌉	1.07

^a^ Number average molecular weight; ^b^ weighted average molecular weight; ^c^ polydispersity index.

## Data Availability

The data are available from the corresponding author.
